# Context matters: Immunology meets ecology

**DOI:** 10.1093/discim/kyaf003

**Published:** 2025-03-04

**Authors:** Iris Mair, Kathryn J Else

**Affiliations:** School of Biological Sciences, College of Science and Engineering, Institute of Ecology and Evolution, Institute of Immunology and Infection Research, University of Edinburgh, Edinburgh, UK; School of Biological Sciences, Division of Infection, Immunity & Respiratory Medicine, Lydia Becker Institute of Immunology and Inflammation, University of Manchester, Manchester, UK

As the field of immunology evolves, it is becoming apparent that the immune system is intricately linked to other physiological and external systems with which it needs to integrate. Thus, when energy is limited, or competing needs arise, the principle of allocation dictates that trade-offs must occur. Readers may have come across the rising calls for ‘rewilding immunology’ [1], ‘naturalising mouse models’[2], or simply ‘dirty mice’, to better mimic the multiple influencers of the immune system. One of our most common immunological models—the laboratory mouse—faces the translational hurdle of how to jump from genetically identical animals living in very controlled conditions to the complex, ever-changing environments that genetically diverse humans and other animals are found in. ‘Ecoimmunology’ combines the two well-established, and seemingly disparate, disciplines of ecology with immunology. Whilst ecology explores uncontrolled variation in a population, immunologists strive to minimize variation to understand mechanisms at play in individuals. At the interface between the two, we discover the exciting research discipline of ecoimmunology, which studies immunology in our multi-variate real world, providing much-needed context to our understanding of immunology [3]. Bringing together researchers from diverse backgrounds using a wide array of study species, technologies, and longitudinal or interventional experimental designs, the results of this approach offer critical insights into how immunity operates outside the confines of the laboratory.

## Defining the unanswered questions

At the heart of ecoimmunology is the recognition that immunity varies between individuals and populations in response to environmental or host-specific changes. For example, how do individuals respond differently to pollution, infectious diseases, or other ecological stressors? What determines this variation, and how does it affect an individual’s fitness and overall health? Beyond individual differences, ecoimmunology also explores immunity at the population level, particularly as environmental factors like climate change, pollution, and urbanization continue to reshape ecosystems. Understanding these shifts is crucial not only for advancing basic immunological knowledge for clinical translation but also for informing conservation efforts and assessing zoonotic disease risks.

## The Special Collection on ‘Ecoimmunology’

This Special Collection in *Discovery Immunology* was motivated by the desire to highlight the importance of context in immunological research. While laboratory immunologists traditionally investigate the effects of one or few factors—like age, sex, or diet—on immune function, there is a growing recognition that more complex biotic and abiotic factors like the microbiome, co-infections, and even tidal rhythms need to be considered when investigating immune function with a translational aspiration to the real world. This collection brings together research that examines these and other factors, offering new insights into how the environment shapes immune responses, and complements the laboratory-based models, which continue to generate new discoveries. With this collection, we want to celebrate the incredible efforts and ingenuity of field-based studies, as well as recognize some of the challenges which come with what one could call a ‘deviation from the standard’.

Just how different are immune systems that operate in multi-variate environments? The phenotypic analyses of the wild mouse bone marrow compartment conducted by Muir *et al.* [4]. revealed that, compared to laboratory mice, the bone marrow of wild mice is strikingly different, with, for example, increased numbers of plasma cells and a remarkable 5-fold increase in eosinophils.

Tackling the microbiome as a significant influencer of the immune system and thus an important extrinsic variable, Viney *et al.* present a discussion about the main drivers of microbiome evolution [5]. Set against the definition of bottom-up and top-down processes, the article presents a case for IgA binding to bacteria acting as a selective pressure such that IgA-bound bacteria evolve at different rates to those species not bound by IgA.

Whilst it is clear that microbial diversity and composition vary between individuals and populations, another understudied confounder is the presence of several natural infections at once, potentially driving disparate immune responses. As one of the first studies showing that Th1 and Th2 responses can be picked up and correlated with age and parasite burden in a wild mammal population living in their natural environment, Corripio-Miyar *et al*. have performed immunological assays in one of the most remote places of the United Kingdom, the archipelago of St Kilda [6].

Moving from biotic to abiotic environmental factors, another review in this collection addresses the effect of temperature on immunity. Anecdotal evidence links temperature changes with immune response variation but a mechanistic understanding of this link is missing. Leading with the question ‘What is a healthy body temperature?’ Maloney *et al*. explore the temperature-immune axis and how, for example, small temperature changes can alter Th cell polarization towards Th17 [7]. Given that body temperature varies with intrinsic factors such as age and sex, and between tissues, it is not hard to see the need to understand better the impact of temperature on immune responses.

It is only a step from thinking of temperature shifts over time to the daily, seasonal, and indeed lunar cycles in which we live. Circadian rhythms are well-documented within the mammalian immune systems but other biological rhythms have received far less attention. Using freshwater three-spined stickleback and analysing 14 immune-associated genes, Jackson *et al.* present a case for a (small) endogenous tidal rhythm, highlighting a gap in our knowledge about how rhythms other than circadian could influence our immune system [8].

Improving our toolbox for analysing immune phenotypes beyond those only achievable via autopsy would bring significant advancement to our ability to understand drivers of immune states over time. A promising tool for wildlife immunological research is the use of extracellular vesicles (EVs). Espejo and Ezenwa present the current evidence for the key role that EVs play in numerous immunological processes [9]. Given their presence in all bodily fluids and tissues, the high conservation across species, as well as their ability to withstand challenging conditions, EVs offer an exciting new frontier for the field of wildlife immunology. As a cautionary tale, on the other hand, Downie *et al*. present a detailed study comparing serum protein levels and white blood cell quantities as predictors of lymphocyte composition [10]. The authors conclude the need to be careful when using serum biomarkers as proxies for cell type abundance.

In conclusion, this Special Collection on Ecoimmunology highlights the ongoing pioneering work in this upcoming research area, by considering the interplay between immune systems and changing environmental and host factors across a variety of model species and experimental approaches. The complexity of ecoimmunological research, and the ‘noise’ which comes with environmental and host variation, of course, brings with it challenges around appropriate analysis and interpretation. Yet, this noise and complexity is in fact the essence of what the immune system of individuals, populations, and ecosystems experiences across time. By embracing the complexity of ‘naturalised’ or natural systems, ecoimmunology approaches offer a promising framework to better understand immunity in real-world contexts, with important implications for human health, wildlife conservation, and disease management.
